# Nonreciprocal thermoelectric cooling by spatially grading Seebeck coefficient for cooling enhancement

**DOI:** 10.1016/j.xinn.2026.101438

**Published:** 2026-05-27

**Authors:** Han Zhao, Zhiwei Chen, Xinyue Zhang, Shuxian Zhang, Xuemei Wang, Shaoqin Wang, Minghao Xue, Jiye Zhang, Wen Li, Jun Luo, Yanzhong Pei

**Affiliations:** 1 Interdisciplinary Materials Research Center, School of Materials Science and Engineering, Tongji University, 4800 Caoan Rd., Shanghai 201804, China; 2 School of Materials Science and Engineering, Shanghai University, 99 Shangda Road, Shanghai 200444, China

**Keywords:** thermoelectric cooling, Peltier effect, Thomson effect, graded materials

## Abstract

Within a unified thermoelectric description, the Peltier and Thomson effects essentially involve changes in electronic entropy along the electron transport pathway, manifested as a spatial gradient in the Seebeck coefficient (∇_*x*_*S*). The former originates from the chemical potential gradient and the latter from the temperature gradient. However, the Thomson effect is usually negligible for a conventional thermoelectric device because ∇_*x*_*S* across the operating temperature range is typically small. This work proposes an extended figure of merit (*z*_extended_) considering the electronic entropy change throughout the bulk thermoelectric material, which originates from the total ∇*S* with respect to all relevant independent variables, including but not limited to temperature (*T*), chemical potential (*μ*), magnetic field (*B*), and others. Experimentally, the electronic entropy change was realized by spatially grading chemical potential in a compositionally graded n-type Bi_2_Te_2.7_Se_0.3_ single-leg device, resulting in nonreciprocity in thermoelectric cooling with respect to the electric current direction. By aligning the direction of ∇_*x*_*μ* with that of the electric current, nonreciprocal thermoelectric cooling yields a 10% improvement in the maximum temperature span (Δ*T*_max_) compared to the cooler without nonreciprocity. Moreover, this work reveals a trade-off between maximizing additional heat absorption and preserving the optimal *zT*, providing a refined approach to cooling enhancement using graded materials. The strategy of nonreciprocal thermoelectric cooling can, in principle, be applied to any dopable material, introducing new degrees of freedom to improve thermoelectric cooling.

## Public summary

• An extended figure of merit was proposed considering the total electronic entropy change.
• A compositionally graded n-type Bi_2_Te_2_._7_Se_0.3_ was grown to achieve spatial grading of the Seebeck coefficient.
• Nonreciprocal thermoelectric cooling and a 10% enhancement were realized in the as-grown single-leg device.
• The strategy can be applied to any dopable materials, providing a new pathway for thermoelectric cooling.

## Graphical abstract

**Figure:**
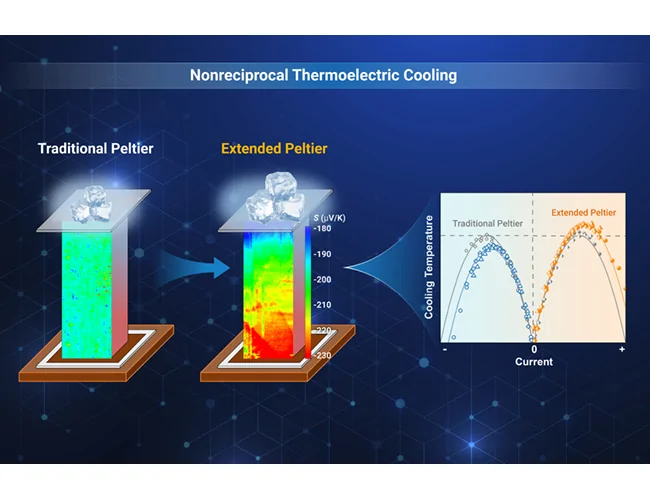

## Introduction

Thermoelectric coolers^1^ are expected to revolutionize thermal energy management by offering a solid-state and eco-friendly alternative that does not require hazardous refrigerants.^2^ Utilizing charge carriers (e.g., electrons^3^ and ions^4^) as working media, thermoelectric coolers enable direct energy conversion between heat and electricity through the manipulation of electronic entropy flow.^5^^,^^6^^,^^7^ For a given electric current density, the entropy current density primarily manifests as the Seebeck coefficient. William Thomson established the Kelvin relations, which thermodynamically relate entropy and heat during thermoelectric processes.^8^ The latter is usually described as the Peltier effect associated with the passage of electrons from one material to another, due to the chemical potential difference at junctions.

Over the past decades, research on thermoelectric cooling has focused on the Peltier effect.^9^^,^^10^ The maximum temperature span (Δ*T*_max_ = *ZT*_c_^2^/2, where the subscript c stands for the cold end) of a Peltier cooler is determined by the dimensionless figure of merit (*ZT* is used for devices when considering the practical heat losses from heat leakages, interfacial issues, etc., and *zT* is used for materials when ignoring such losses). Materials’ *zT* is usually expressed as *zT* = *S*^2^*T*/*ρκ*, where *S*, *ρ*, *T*, and *κ* are the Seebeck coefficient, electrical resistivity, absolute temperature, and thermal conductivity, respectively.^11^ The inherent trade-off among the three physical parameters (*S*, *ρ*, and *κ*)^10^^,^^12^^,^^13^ makes it challenging to enhance materials’ *zT*. Therefore, several strategies, such as manipulating energy bands^14^^,^^15^ and microstructures,^16^ have been developed to decouple these physical parameters.

The Thomson effect, an integral component of the Kelvin relations, offers an alternative thermoelectric cooling mechanism by enabling heat absorption throughout the bulk thermoelectric material.^17^ Theoretical studies^18^^,^^19^ suggest that for materials with identical *zT*, the Thomson effect could potentially double the achievable temperature span (Δ*T*) compared to the conventional Peltier effect. This large Thomson span requires not only a large material *zT* but also a large Thomson coefficient (*τ*).

In previous studies, the Thomson and its related effects^20^^,^^21^^,^^22^ are dominated by the partial derivative of the Seebeck coefficient with respect to temperature (Figure 1A), which is linked to the electronic band structure. The narrow-defined Thomson coefficient is

(Equation 1) $$\tau =T\frac{\partial S}{\partial T}=T\frac{2\frac{k}{e}\partial \left(\frac{\int_{0}^{\infty }\frac{x}{1+\exp\left[x-\frac{\mu }{kT}\right]}dx}{\int_{0}^{\infty }\frac{1}{1+\exp\left[x-\frac{\mu }{kT}\right]}dx}-\frac{\mu }{kT}\right)}{\partial T}=-\frac{k}{e}\frac{\mu }{kT}\left(1+\frac{2\;\exp\left[\frac{\mu }{kT}\right]\sum_{i=1}^{\infty }\frac{{-\exp\left[\frac{\mu }{kT}\right]}^{i}}{k^{2}}}{\left(1+\exp\left[\frac{\mu }{kT}\right]\right)\ln\;{\left[1+\exp\left[\frac{\mu }{kT}\right]\right]}^{2}}\right)\text{,}$$

where *k*, *e*, and *μ* are the Boltzmann constant, electron charge, and chemical potential, respectively. The expression of the Seebeck coefficient is derived from the Boltzmann transport theory based on a single parabolic band with acoustic phonon scattering.^23^ Thus, the current understanding is that a large *τ* requires a rapid increase in Seebeck coefficient with increasing temperature,^24^^,^^25^^,^^26^^,^^27^ and recent experimental studies realized it by leveraging electron-correlated phase transitions, including antiferromagnetic-ferromagnetic transition,^25^ Kondo lattice effects,^26^ and charge density wave transition.^28^ However, these mechanisms exist in specific materials whose *zT* values are much lower than those of classical thermoelectric materials such as Bi_2_Te_3_-based alloys,^29^ which limits the widespread application of the Thomson effect for cooling.^30^^,^^31^^,^^32^ Therefore, the key challenge to developing efficient Peltier-Thomson-combined thermoelectric coolers is to realize a large electronic entropy change in materials that already have a high *zT*.

**Figure 1:**
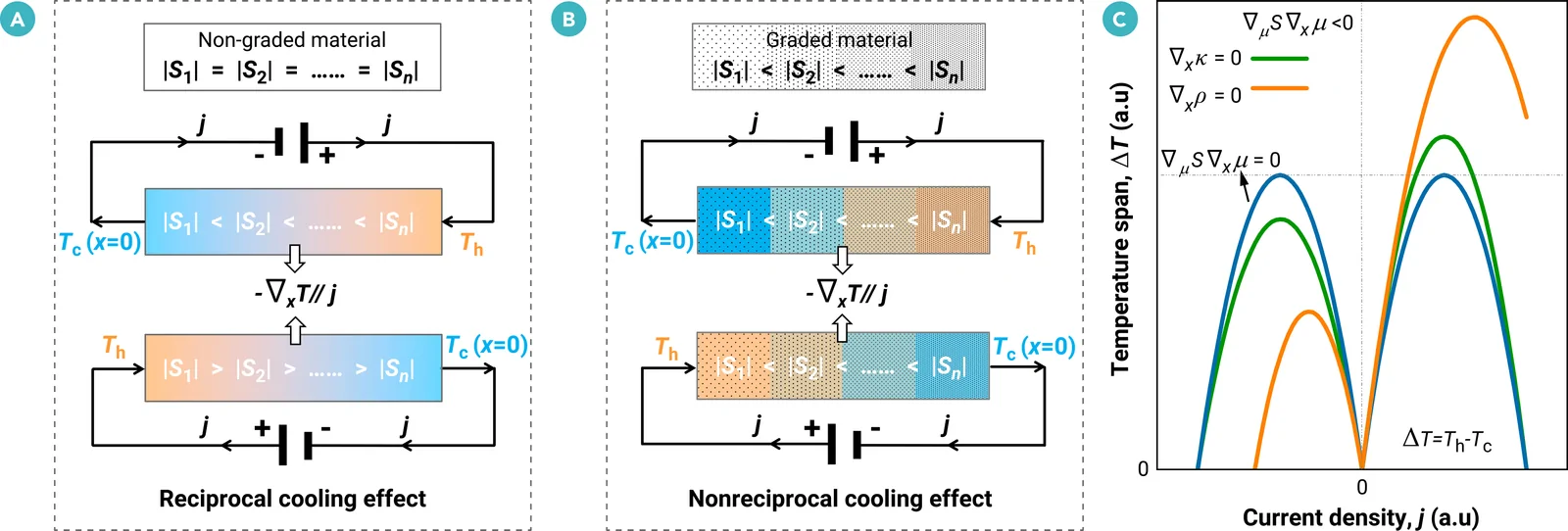
Schematic of the thermoelectric cooling effects without and with nonreciprocity (A and B) Taking an n-type semiconductor as an example, the transporting electrons increase the electronic entropy (manifested as the Seebeck coefficient) for additional heat absorption along the temperature gradient (∇_*x*_*T*) direction in a non-graded material (A) or along the Seebeck gradient (∇_*x*_*S*) direction due to a chemical potential gradient (∇_*x*_*μ*) in a graded material (B). (C) Current density (*j*)-dependent temperature span (Δ*T*) with or without ∇_*μ*_*S*∇_*x*_*μ*: the maximum Δ*T* increases when *j* is antiparallel to ∇_*μ*_*S*∇_*x*_*μ* but decreases when *j* is parallel to ∇_*μ*_*S*∇_*x*_*μ*, showing the nonreciprocal effect in thermoelectric cooling.

Given that the change in electronic entropy throughout the electron transport pathway (∇_*x*_*S*) is the essence of thermoelectric cooling, this work realized cooling enhancement in conventional n-type Bi_2_Te_3_ utilizing the generalized thermoelectric cooling effect, which is expressed as

(Equation 2) $$\nabla_{x}S=\frac{\partial S}{\partial T}\frac{\partial T}{\partial x}+\frac{\partial S}{\partial \mu }\frac{\partial \mu }{\partial x}+\frac{\partial S}{\partial B}\frac{\partial B}{\partial x}+\ldots \text{,}$$

where the total differential of *S* is the sum of its partial differentials with respect to all independent variables, including temperature (*T*), chemical potential (*μ*), magnetic field (*B*), and others. Note that the first term on the right-hand side is the simplification presuming the Thomson effect (as given by Equation 1) and the second term is the simplification presuming the Peltier effect.

## Materials and methods

### Synthesis

In this work, high-purity bismuth (Bi; 99.99 wt %), tellurium (Te; 99.99 wt %), selenium (Se; 99.99 wt %), and tellurium iodide (TeI_4_; 99.99%) were accurately weighed according to the stoichiometric ratio of Bi_2_Te_2.7-*δ*_Se_0.3_I_*δ*_. The raw materials were mixed and placed into a quartz tube with an inner diameter of 12 mm and a 60° tip angle, which was sealed under vacuum. The quartz tube was suspended in a vertical gradient furnace for crystal growth (Figure S1). The temperature settings employed during the material growth process are shown in Figure S1C. The gradient furnace was heated from 300 to 1,173 K at a rate of 145 K/h, then maintained at 1,173 K for 6 h to pre-melt the raw materials. The furnace temperature was then lowered to 893 K (a temperature above the melting point of the material) over 2 h, and thereafter it was lowered slowly at a rate of 2 K/h until each region of the ingot was below the melting point of the material. The as-grown ingots were then cut into bar-shaped samples using a wire cutting machine for subsequent characterization and measurement. The dimensions of the commercial legs and graded legs are as follows: 39.4 × 4.4 × 3.47 mm^3^ for commercial 1, 39.72 × 4.47 × 3.74 mm^3^ for commercial 2, 38.87 × 4.43 × 2.78 mm^3^ for device 1, 38.8 × 4.42 × 2.7 mm^3^ for device 2, and 38.83 × 4.42 × 2.78 mm^3^ for device 3.

### Characterization

The phase compositions of the materials were characterized by X-ray diffraction (XRD; DX-2700) and a scanning electron microscope (SEM; Phenom Pro) equipped with an energy-dispersive spectrometer (EDS). The as-grown materials are well oriented along the (00L) plane (Figure S2A) and have a uniform distribution of elements (Figure S2B). The spatial Seebeck profile of the samples was measured using commercial equipment, the Potential Seebeck Microprobe (PSM II, Panco, Germany). The schematic diagram of the measurement is illustrated in Figure S3. The sample under test was firmly fixed on the sample stage using a dedicated fixture. The measurement probe featured precise three-dimensional translational and positioning capabilities, enabling accurate characterization of the two-dimensional distribution of the Seebeck coefficient across the sample surface. The positioning accuracy of the equipment is 1 μm in both directions, and the measurement error for the Seebeck coefficient is <3% for semiconductors.

### Harman method

The device *ZT* and cooling performance were measured using the Harman method. A schematic illustration of the measurement setup is provided in Figure S4. The measurements were conducted in a vacuum chamber to minimize thermal radiation and convection. One end of the single-leg device (*T*_h_ end) was attached to a copper heat sink with thermal grease for efficient heat dissipation. The heat sink was water cooled and temperature controlled via a programmed temperature controller. Copper electrodes and current-carrying (CC) wires (copper, AWG 18) were soldered to both ends of the single-leg device, while voltage-measuring (VM) wires (copper, AWG 36) and thermocouples (K-type, AWG 36, Omega Engineering) were attached using silver epoxy. A six-and-a-half-digit digital multimeter (TH1963, Tonghui) was used to guarantee accurate voltage and temperature readings. Voltage and temperature were simultaneously recorded as functions of time. When the current was zero, both the voltage and temperature span Δ*T* were zero. Given that the electrical response is much faster than the thermal response, the isothermal voltage *V*_i_ can be obtained right after current application, before a Δ*T* is able to develop within the device. With prolonged current application, the device forms a stable Δ*T*, and the measured voltage (adiabatic *V*_a_) includes an additional thermoelectric voltage induced by this Δ*T*. The Harman *ZT* is then derived as *ZT* = *V*_a_/*V*_i_ − 1. Note that the cold-end CC wire can be equivalent to the other leg of a *π*-type thermoelectric couple to quantify the attenuation effect of its heat loss on the device’s *ZT* and Δ*T*. This means that the *ZT* and Δ*T* directly measured by the Harman method actually include the effects of the heat loss from the cold-end CC wire.

## Results and discussion

### Derivation of extended figure of merit

Introducing a chemical potential gradient (∇_*x*_*μ*) can spatially modulate the Seebeck coefficient distribution in materials, realizing a large, spatially graded Seebeck coefficient (∇_*x*_*S* ≈ ∇_*μ*_*S*∇_*x*_*μ*; note that ∇_*μ*_*S* is usually plotted as a Pisarenko line). As illustrated in Figure 1B, when a ∇_*x*_*S* exists in a material, the heat flux equations under local thermal equilibrium can be expressed as

(Equation 3a) $$-\kappa \nabla_{x}T+jTS=q\;\text{and}$$

(Equation 3b) $$\kappa \nabla_{x}^{2}T+\rho j^{2}=jT\nabla_{x}S=jT\left(\nabla_{T}S\nabla_{x}T+\nabla_{\mu }S\nabla_{x}\mu \right)\text{.}$$

It is noteworthy that the Peltier heat (*jTS*) at the cold end (*T*_c_) induces a temperature gradient (∇_*x*_*T*), which is always antiparallel to the current density (*j*) direction for an n-type semiconductor. Thus, the ∇_*x*_*T*-induced Thomson heat (*jT*∇_*x*_*S*) is reciprocal. That is, the temperature span remains independent of the current direction (Figure 1A). However, since the direction of the chemical potential gradient (∇_*x*_*μ*) is independent of *j*, the extended Peltier heat exhibits nonreciprocal characteristics (Figure 1B). Here, the direction of increasing absolute value of the Seebeck coefficient is defined as the positive direction for the spatial gradient of Seebeck (∇_*x*_*S*). For an n-type semiconductor, when *j* is antiparallel to the spatial gradient of Seebeck (∇_*x*_*S*) due to the chemical potential gradient (∇_*x*_*μ*), the electrons experience an increase in electronic entropy along the transport pathway, leading to heat absorption and providing additional thermoelectric cooling. Conversely, when *j* is parallel to that of ∇_*μ*_*S*∇_*x*_*μ*, it produces heating (heat emission).

To provide an analytical form of the temperature distribution, we assume that (1) there is no spatial gradient of the thermal conductivity (∇_*x*_*κ* = 0), (2) the resistivity exhibits a gradient (∇_*x*_*ρ*) to keep *dz*/*dx* = (2*S*/*ρκ*)∇_*x*_*S* − (*S*^2^/*ρ*^2^*κ*)∇_*x*_*ρ* = 0, and (3) ∇_*x*_*S* is independent of *x*. Under these approximations, Equation 3b is rewritten as

(Equation 4) $$\kappa \nabla_{x}^{2}T-jT\nabla_{x}S+{\left(x\nabla_{x}S+S_{x=0}\right)}^{2}j^{2}/z\kappa =0\text{.}$$

This equation is a second-order linear non-homogeneous ordinary differential equation. The general solution (in analytical form) can be obtained by combining the homogeneous solution with a particular solution. Let *a* = −*κj*∇_*x*_*S* and *b* = *j*^2^/(*κ*^2^z). Equation 4 becomes

(Equation 5) $$\nabla_{x}^{2}T+aT=-b{\left(x\nabla_{x}S+S_{x=0}\right)}^{2}\text{.}$$

Since ∇_*x*_*S* ≠ 0, we assume that *j*∇_*x*_*S* > 0 (i.e., *a* < 0) and define *α* = (−*a*)^0.5^ = (*κj*∇_*x*_*S*)^0.5^, leading to the hyperbolic functions of the homogeneous solution,

(Equation 6) $${\;T}_{\text{homogeneous}}=C_{1}\;\cosh\left[\alpha x\right]+C_{2}\;\sinh\left[\alpha x\right]\text{.}$$

Then, we assume a particular solution of the form *T*_particular_ = *P* + *Qx* + *Rx*^2^. Substituting this into Equation 5, we have

(Equation 7) $$2R+a\left(P+Qx+Rx^{2}\right)=-b{\left(x\nabla_{x}S+S_{x=0}\right)}^{2}\text{.}$$

Matching the coefficients yields

(Equation 8) $$R=\frac{j\nabla_{x}S}{\kappa z},Q=\frac{2jS_{x=0}}{\kappa z},P=\frac{jS_{x=0}^{2}}{\kappa z\nabla_{x}S}+\frac{2}{z}\text{.}$$

Consequently, the general solution is *T* = *T*_homogeneous_ + *T*_particular_. Applying the boundary conditions to determine the integration constants. From *T*_*x*=0_ = *T*_c_,

(Equation 9) $$C_{1}+P=T_{c}\to \;C_{1}=T_{c}-\frac{jS_{x=0}^{2}}{\kappa z\nabla_{x}S}-\frac{2}{z}\text{.}$$

From *q*_*x*=0_ = −*κ*∇_*x*_*T* + *jT*_c_*S*_*x*=0_ = 0,

(Equation 10) $$\nabla_{x}T_{x=0}=\alpha C_{2}+Q\to \;C_{2}=\frac{jS_{x=0}}{\kappa a}\left(T_{c}-\frac{2}{z}\right)\text{.}$$

Substituting *C*_1_, *C*_2_, and *T*_particular_ into the general solution and simplifying, we obtain the final analytical expression for *T*(*x*), as given by Equation 11a. Solving the extremum problem of this equation yields a transcendental equation, of which the solution (the maximum temperature span, Δ*T*_max_) cannot be expressed in an analytical form. We have to adopt a strong approximation of constant *T*∇_*x*_*S*, which is more valid for considering an infinitesimal section of the thermoelectric leg. Equation 11a can be rewritten in polynomial form, as given by Equation 11b:

(Equation 11a) $$T\left(x\right)=\frac{j{\left(x\nabla_{x}S+S_{x=0}\right)}^{2}+2\kappa \nabla_{x}S+\left(-jS_{x=0}^{2}+\kappa \left(zT_{c}-2\right)\nabla_{x}S\right)\cosh\left[x\frac{\sqrt{j\nabla_{x}S}}{\sqrt{\kappa }}\right]+\sqrt{j\nabla_{x}S}S_{x=0}\left(zT_{c}-2\right)\sqrt{\kappa }\sinh\left[x\frac{\sqrt{j\nabla_{x}S}}{\sqrt{\kappa }}\right]}{z\kappa \nabla_{x}S}\;\text{and}$$

(Equation 11b) $$T\left(x\right)=\frac{-6j^{2}S_{x=0}^{2}x^{2}-4\nabla_{x}Sj^{2}S_{x=0}x^{3}-{\nabla_{x}S}^{2}j^{2}x^{4}+12jS_{x=0}\kappa zT_{c}x+6\nabla_{x}S\kappa zTjx^{2}+12zT_{c}\kappa^{2}}{12z\kappa^{2}}\text{.}$$

Please recall that ∇_*x*_*S* is the sum of the partial differentials with respect to all independent variables. The ∇_*x*_*S* in Equations 11a and 11b can be replaced by either ∇_*T*_*S*∇_*x*_*T* or ∇_*μ*_*S*∇_*x*_*μ*, if one of the spatial gradients is dominant. With *S*_c_ and *S*_h_ being the Seebeck coefficients at the cold and hot sides, the extremum problem of Equation 11b yields a simplified expression for estimating Δ*T*_max_, as given by Equation 12a.

(Equation 12a) $$\Delta T_{\max}\approx \frac{zT_{c}^{2}}{2}\frac{{\;3\left(\left(S_{h}-S_{c}\right)+2S_{c}\right)}^{2}}{2\left({\left(S_{h}-S_{c}\right)}^{2}+4\left(S_{h}-S_{c}\right)S_{c}+6{S_{c}}^{2}\right)\;}=\frac{zT_{c}^{2}}{2}\frac{{\;3\left(\left(\frac{S_{h}}{S_{c}}-1\right)+2\right)}^{2}}{2\left({\left(\frac{S_{h}}{S_{c}}-1\right)}^{2}+4\left(\frac{S_{h}}{S_{c}}-1\right)+6\right)\;}=\frac{z_{\text{extended}}T_{c}^{2}}{2}\text{.}$$

Similarly, Equation 6 can be derived to Equation 12b when replacing the approximation ∇_*x*_*κ* = 0 by ∇_*x*_*ρ* = 0.

(Equation 12b) $$\Delta T_{\max}\approx \frac{zT_{c}^{2}}{2}\frac{{\;\left(2\left(S_{h}/S_{c}-1\right)+\ln\left[\frac{S_{c}}{S_{h}}\right]\right)}^{2}}{2\left(\left(S_{h}/S_{c}-1\right)+\ln\left[\frac{S_{c}}{S_{h}}\right]\right)\;}=\frac{z_{\text{extended}}T_{c}^{2}}{2}$$

In this work, Equations 12a and 12b aim to conveniently compare the Δ*T*_max_ for the generalized thermoelectric cooling effect with that for traditional Peltier (Δ*T*_max_ = *zT*_c_^2^/2). The Δ*T*_max_ itself can be obtained by plotting the Δ*T* vs. *j* based on Equation 11a, without using the strong approximation of constant *T*∇_*x*_*S.* The approximation of ∇_*x*_*κ* = 0 is more valid if the total thermal conductivity (*κ*) is dominated by the lattice component (∇_*x*_*κ* = ∇_*μ*_*κ*_L_∇_*x*_*μ*), as the change of the chemical potential would barely affect the lattice thermal conductivity (∇_*μ*_*κ*_L_ ≈ 0). On the other hand, the approximation of ∇_*x*_*ρ* = 0 is more valid if the band effective mass is small (like Bi_2_Te_3_ here), as ∇_*μ*_*ρ* is rather small. These two approximations are believed to give the upper and lower boundaries for a given ∇_*x*_*S*. The experimental data of cooling performance with arbitrary ∇_*x*_*κ* and ∇_*x*_*ρ* would lie between the modeling predictions based on these two boundaries.

Figure 1C models the temperature span (Δ*T*) as a function of the current density for the different approximations (∇_*x*_*κ* = 0 or ∇_*x*_*ρ* = 0). Overall, it is found that the nonreciprocal effect of Δ*T*_max_ stems from the direction of current density in the presence of ∇_*x*_*μ*. When the electrons move from the cold end to the hot end, Δ*T*_max_ increases for *j* > 0 but decreases for *j* < 0. There are two types of asymmetries: the optimal current and the temperature span. Firstly, the former is proportional to the local Seebeck coefficient over the local resistivity. When ∇_*x*_*κ* = 0, the existence of ∇_*x*_*ρ* partially balances the effect of ∇_*μ*_*S*∇_*x*_*μ* on the local optimal current, which reduces the asymmetry between *j* > 0 and *j* < 0. On the other hand, when ∇_*x*_*ρ* = 0, the existence of ∇_*x*_*κ* would affect the local heat flux via *q* = −*κ*∇_*x*_*T* + *jTS*. The boundary condition *q*_*x*=0_ = −*κ*∇_*x*_*T*_*x*=0_ + *jT*_c_*S*_*x*=0_ = 0 holds for both *j* > 0 and *j* < 0. This eventually leads to substantial differences in total cooling power and, consequently, the temperature span, which enhances the asymmetry. These results theoretically confirm the proposed physical scenarios, where the generalized thermoelectric cooling effect is the result of the electronic entropy increase or decrease throughout the bulk thermoelectric material due to chemical potential and/or temperature gradients. More importantly, the thermoelectric figure of merit can be rewritten as *z*_extended_ in Equations 12a and 12b, considering the contribution of the generalized thermoelectric cooling effect.

### Device *ZT* and cooling performance

We synthesized a compositionally graded n-type Bi_2_Te_2.7_Se_0.3_ using iodine as a dopant to experimentally demonstrate practical cooling utilizing the generalized thermoelectric cooling effect. Since the compositional gradient primarily changes the chemical potential gradient, the following discussion mainly focuses on the extended Peltier effect. In this work, the key to fabricating graded thermoelectric devices/materials lies in selecting an appropriate doping concentration and precisely controlling it. As shown in Figure S5, when the iodine doping concentration reaches *δ* ≥ 0.005 for Bi_2_Te_2.7-*δ*_Se_0.3_I_*δ*_, the obtained materials are nearly homogeneous. Considering both a large Seebeck coefficient difference (Δ*S*) and a Seebeck coefficient (*S*) range enabling high thermoelectric performance, an iodine doping concentration of *δ* ≈ 0.0035 is identified as the optimal choice for the starting materials in this work. To further quantify the contributions to Δ*T*_max_, we designed the experimental configuration shown in Figures 1B and S4, in which both the traditional and extended Peltier effect coexist under applied current. Specifically, for an n-type Bi_2_Te_2.7_Se_0.3_, traditional Peltier cooling occurs when electrons flow into the thermoelectric material, while heating occurs when electrons flow out, independent of the sign of ∇_*x*_*μ*. By switching the direction of current, we get two different configurations: the *j* is antiparallel or parallel to that of ∇_*μ*_*S*∇_*x*_*μ*. The former is the synergistic mode as “P_trad._ + P_ext._” and the latter is the compensatory mode as “P_trad._ − P_ext._” By comparing the data measured in these two configurations, the individual contribution can be effectively decoupled, enabling quantitative estimation of the extended Peltier contribution to the overall cooling performance.

Figures 2A and 2C present the Seebeck coefficient mapping results of the commercial extruded n-type Bi_2_Te_2.7_Se_0.3_ (Xmeritan) and the compositionally graded n-type Bi_2_Te_2.6965_Se_0.3_I_0.0035_ synthesized in this work. The Seebeck coefficient of the compositionally graded sample exhibits a near-linear spatial variation along the *x* direction, reaching a maximum difference (Δ*S*_*μ*_) of ∼50 μVK^−1^. The commercial sample shows a homogeneous Seebeck distribution. Several bar-shaped single-leg devices, including both commercial and as-grown graded ones, were then fabricated, and their cooling performance was measured using the Harman method.^33^

**Figure 2:**
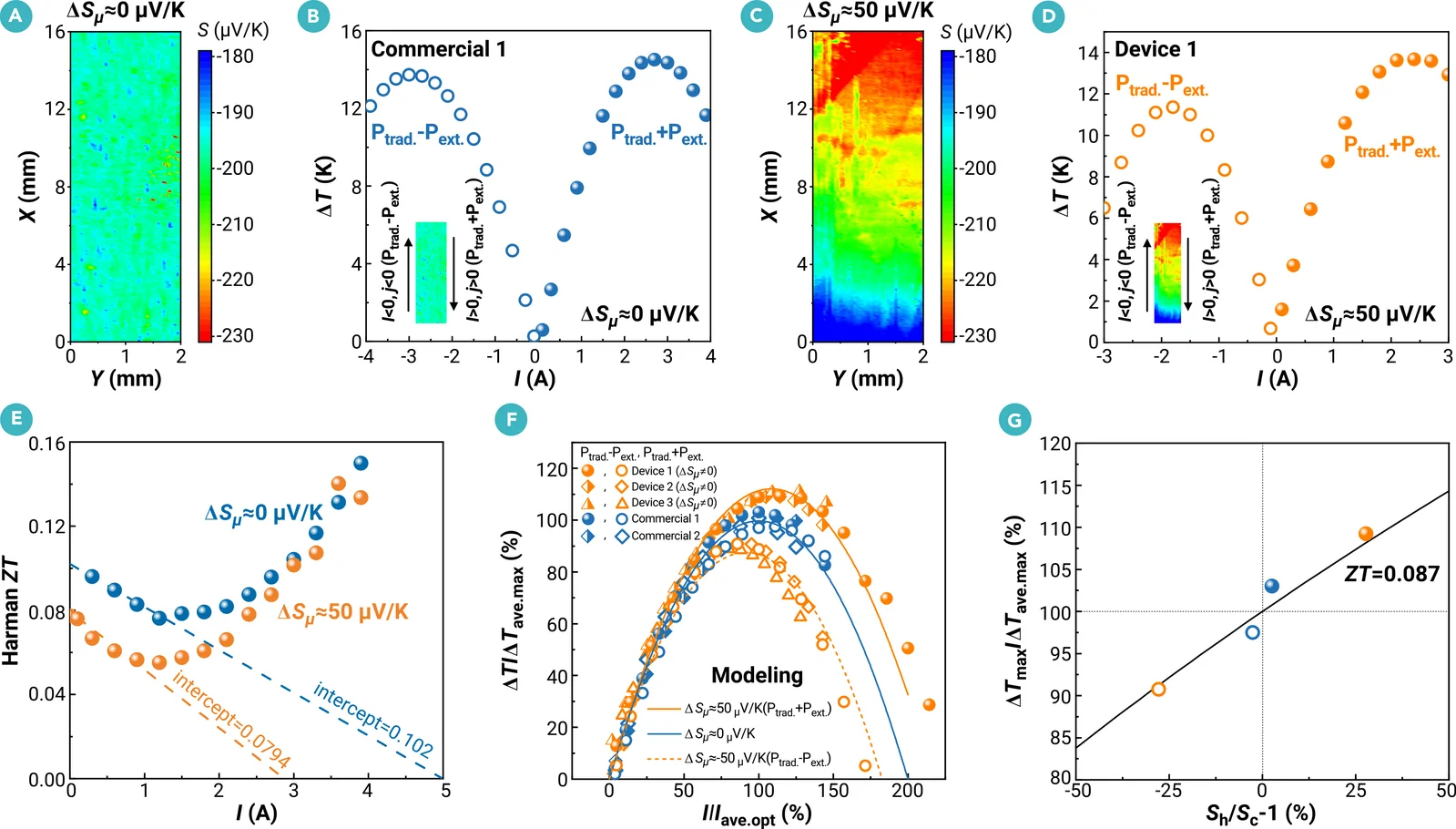
Nonreciprocity in cooling performance at *T*_h_ = 300 K (A–D) Seebeck coefficient mapping results (A and C) and current-dependent temperature span (B and D) for commercial extruded and compositionally graded Bi_2_Te_2.7_Se_0.3_ samples. (E) Harman *ZT*. (F and G) Normalized analyses of temperature span for current (F) and spatial gradient of Seebeck coefficient (G), where the solid and open circles refer to the P_trad._ + P_ext._ and P_trad._ − P_ext._ modes, respectively. Both theory and experiments demonstrate that the extended Peltier effect can enhance thermoelectric cooling performance without improving *ZT*.

The current (*I*)-dependent temperature spans (Δ*T*) at *T*_hot_ = 300 K for both P_trad._ + P_ext._ and P_trad._ − P_ext._ modes are shown in Figures 2B and 2D, the insets of which show the current directions. Here, Δ*T* is defined as the temperature difference between the bottom surface (hot end, heat-sink connected) and top surface (cold end). The voltage, resistance, resistivity, and Δ*T* data recorded as a function of time in the P_trad._ + P_ext._ mode are shown in Figures S6 and S7. To verify the reproducibility of the results, two commercial single legs (labeled commercial 1 and commercial 2) and three graded single legs (labeled devices 1–3) were measured, as shown in Figures S8 and S9. Note that when the measurement duration was sufficiently long for the temperature difference to reach steady state, the temperature fluctuation detected by the system was basically no more than 0.05 K. In addition, the test results for a commercial 7-pair Bi_2_Te_3_ module obtained by the present test system (Δ*T*_max_ of 63 K at *T*_h_ of 300 K; Figure S10) are consistent with the performance specifications, thus verifying the reliability of the cooling results from this system.

For single-leg thermoelectric devices, the cold-end CC wire can be treated as equivalent to the other leg of a π-type thermoelectric couple to quantify the attenuation effect of its heat loss on the device’s *ZT* and Δ*T*. In this way, the theoretical device *ZT* can be calculated via *ZT* = (*S*_TE_ − *S*_lead_)2/[(*Κ*_TE_ + *Κ*_lead_)(*R*_TE_ + *R*_lead_)]*T*, in which *Κ* and *R* represent thermal conductance and electrical resistance and the subscripts TE and lead refer to the thermoelectric material and the electrical lead (cold-end CC wire here). Taking the commercial n-type leg as an example (with a material *zT* of 0.86 and dimensions of 39.4 × 4.4 × 3.47 mm^3^), at room temperature, *S*_TE_ = −201 μV/K, *R*_TE_ = 0.024 Ω, and *Κ*_TE_ = 5.9 × 10^−4^ W/K. The parameters for the CC wire (copper, AWG18, length: 100 mm) are as follows: *S*_lead_ = +2 μV/K, *R*_lead_ = 0.002 Ω, and *Κ*_lead_ = 0.0033 W/K. The calculated device *ZT* is ∼0.123, corresponding to a theoretical Δ*T*_max_ of ∼18 K. The calculated results closely match the *ZT* experimentally measured using the Harman method (Figure 2E) and the measured Δ*T*_max_ of ∼15 K (Figure 2B), consistent with the prediction for a traditional Peltier cooler (Δ*T*_max_ = *ZT*_c_^2^/2). This means that the directly measured *ZT* and Δ*T* here actually include the primary heat loss from the cold-end CC wire. This is the reason why, even using a commercial material, the achieved Δ*T*_max_ of ∼15 K for a single leg is typically smaller than that reported in commercial modules (Δ*T*_max_ of ∼65 K).^34^

Nevertheless, the commercial and compositionally graded Bi_2_Te_2.7_Se_0.3_ used in this work show comparable optimal current (*I*_opt_) and Δ*T*_max_. Obvious nonreciprocal Δ*T* was observed in compositionally graded Bi_2_Te_2.7_Se_0.3_, due to the existence of a spatially graded Seebeck coefficient (Figure 2D). It is worth noting that while some works have reported the non-reciprocity of certain electrical and thermal transport properties in thermoelectric materials,^35^^,^^36^^,^^37^^,^^38^ the non-reciprocity of cooling properties has not yet been reported.

Both P_trad._ + P_ext._ and P_trad._ − P_ext._ modes are carried out to decouple the respective contributions of the traditional Peltier and the extended Peltier effects to Δ*T*. To facilitate a more intuitive comparison, Δ*T* and *I* are normalized by their respective average values of Δ*T*_max_ and *I*_opt_ obtained from P + T and P − T modes (Figure 2F), where Δ*T*_ave.max_ = (Δ*T*_max,Ptrad.+Pext._ + Δ*T*_max,Ptrad.−Pext._)/2 and *I*_ave.opt_ = (*I*_opt, Ptrad.+Pext._ + *I*_opt, Ptrad.−Pext._)/2. All experimental data exhibit good agreement with the predictions. For a commercial single leg with Δ*S*_*μ*_ ≈ 0, the opposite current direction barely affects the Δ*T*, confirming the absence of extended Peltier contribution. The minor discrepancies from the theoretical values (within 3%) in Figure 2F are mainly due to slight variations in heat transfer between the device and the heat sink during the current-direction switch.

For compositionally graded Bi_2_Te_2.7_Se_0.3_, the P_trad._ + P_ext._ mode achieves a 20% higher Δ*T*_max_ compared to the P_trad._ − P_ext._ mode, indicating that the extended Peltier effect provides an additional 10% enhancement beyond the traditional Peltier effect. Meanwhile, a shift in *I*_opt_ between the P_trad._ + P_ext._ and P_trad._ − P_ext._ modes was observed in a compositionally graded sample (Figure 2F). This shift indicates that the local heat flux is changed when the current direction is reversed. This suggests a non-zero ∇_*x*_*κ* in the compositionally graded material, as the local heat flux at the cold side depends on the current direction and the temperature profile itself when a Seebeck gradient is present.

Equations 12a and 12b reveal that the *S*_h_/*S*_c_ ratio is the key parameter determining Δ*T*_max_ enhancement, as experimentally validated in Figure 2G. This conclusion is consistent with the analysis reported in the literature.^39^^,^^40^ In commercial Bi_2_Te_2.7_Se_0.3_, the established temperature span also causes a Seebeck coefficient difference, also known as the Thomson effect induced by a temperature gradient. When achieving a Δ*T*_max_ of 15 K at *T*_h_ = 300 K, the Seebeck coefficient of the hot side (*S*_h_) is 201 μV·K^−1^, and that of the cold side (*S*_c_) is 196 μV·K^−1^. Then, the calculated value of (*S*_h_/*S*_c_ − 1) is approximately 2.55%, as shown in Figure 2G. This explains its negligible contribution in traditional Peltier coolers. On the other hand, the Seebeck coefficient difference induced by the chemical potential gradient can reach up to ∼50 μV·K^−1^, leading to an order-of-magnitude increase in *S*_h_/*S*_c_ − 1 and a 10% improvement in Δ*T*_max_. Although the currently achieved, insufficiently large *S*_h_/*S*_c_ ratio limits the cooling enhancement in this work, a more notable improvement can be expected with a further increase in the spatial Seebeck coefficient gradient. In addition, only single-leg thermoelectric devices were fabricated here as a proof of concept. Assembling the gradient materials into paired π-type modules will lead to a much more obvious enhancement in cooling performance than single-leg devices.

### Graded materials for generalized thermoelectric cooling

In fact, the traditional Peltier and the extended Peltier effects are inherently coexistent in all thermoelectric devices under practical working conditions. It is worth noting that the increase in *S*_h_/*S*_c_ might be accompanied by a decrease in material *zT* due to the deviation of the optimal chemical potential or Seebeck coefficient (Figure 3A). This fundamental compromise between the traditional and extended Peltier effects can be evaluated by the thermoelectric quality factor (*B*),

(Equation 13) $$B=\frac{B_{E}}{\kappa_{L}}T\text{,}$$

where *B*_E_ is the electronic quality factor^23^ and *κ*_L_ is the lattice thermal conductivity. Figure 3A shows the relation between *zT* and the Seebeck coefficient at *B* = 0.33, which is a typical value for commercial Bi_2_Te_2.7_Se_0.3_.

**Figure 3:**
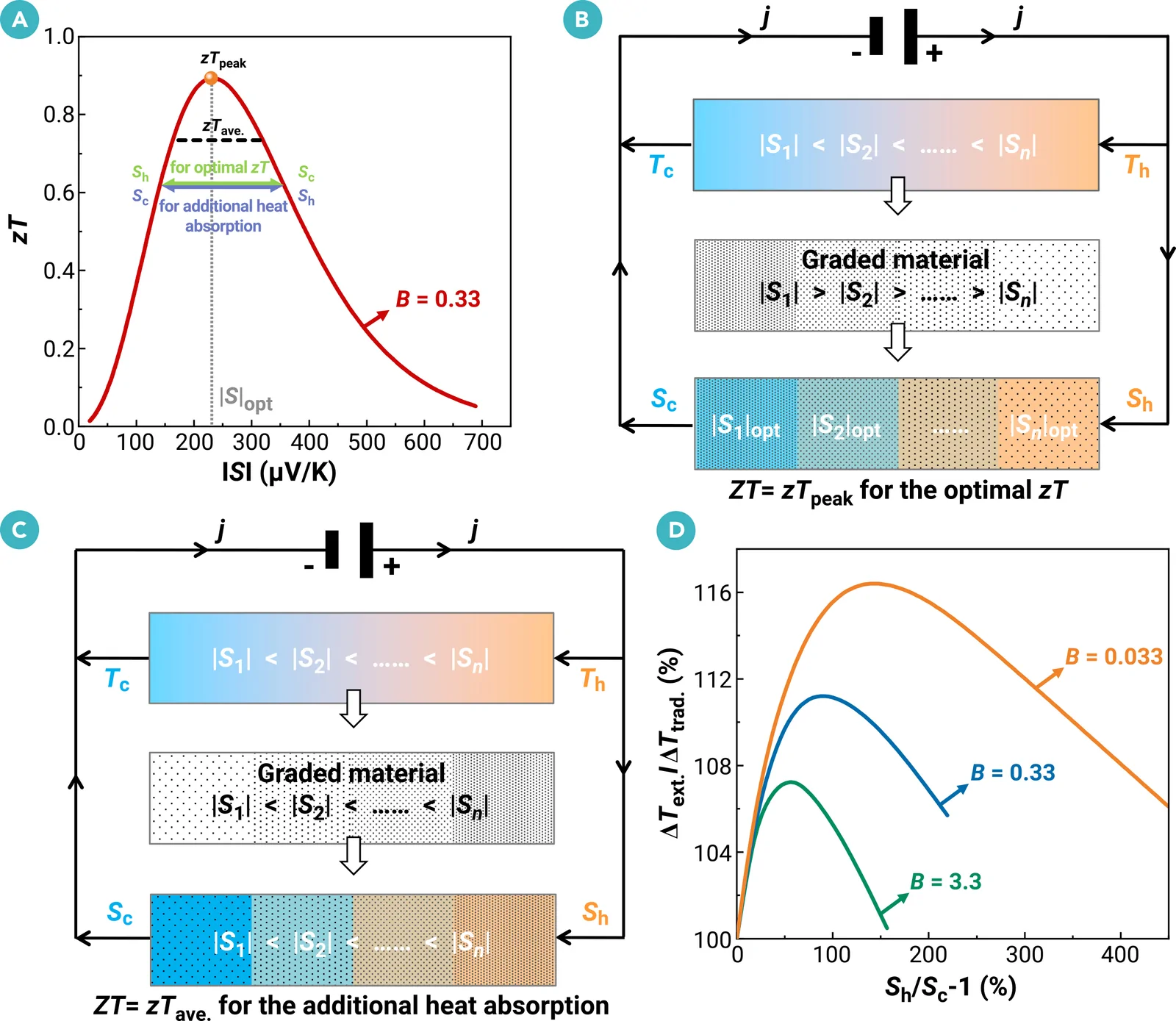
Competition between the optimal *zT* and the additional heat absorption, induced by a composition gradient (A) Seebeck coefficient-dependent material *zT* for a given quality factor (*B* = 0.33) based on a single parabolic band with acoustic phonon scattering. (B and C) Two configurations of graded materials with a spatial gradient Seebeck coefficient (∇_*x*_*S*): ∇_*μ*_*S*∇_*x*_*μ* antiparallel to the temperature gradient ∇_*x*_*T*, ensuring a high material *zT* for the traditional Peltier effect (B), and ∇_*μ*_*S*∇_*x*_*μ* parallel to ∇_*x*_*T*, ensuring the entropy increase for the extended Peltier effect (C). (D) Ratio of Seebeck coefficient (*S*_h_/*S*_c_)-dependent enhancement of cooling temperature due to the extended Peltier effect for different quality factors.

Conceptually, the spatial gradient of the Seebeck coefficient via a graded material can be interpreted either as the optimal *zT* or as the additional heat absorption, depending on how one understands the generalization of thermoelectric phenomena. The most important point is that these two distinct understandings would lead to fundamentally different approaches in leveraging graded materials to enhance cooling performance, as illustrated in Figures 3B and 3C.

For the optimal *zT*, it is expected that the peak *zT* with the optimal Seebeck coefficient (*S*_opt_ in Figure 3B) can always be achieved throughout the thermoelectric leg. For a degenerated semiconductor, the Seebeck coefficient usually decreases upon cooling (∇_*T*_*S* > 0). Thus, the Seebeck coefficient at the cold end will be lower than that at the hot end when a non-graded cooler is working (top, Figure 3B). This deviation from the optimal Seebeck coefficient would degrade the overall device *ZT*. This implies that, for a graded cooler (middle, Figure 3B), the Seebeck coefficient at the cold end should be pre-designed to be higher than the optimal Seebeck coefficient, while that at the hot end should be pre-designed to be lower than the optimal Seebeck coefficient (the chemical potential gradient is antiparallel to the temperature grading, e.g., ∇_*x*_*μ*∇_*x*_*T* < 0). This is the conventional understanding of graded materials in thermoelectric cooling, where the device *ZT* should be designed to be equal to the peak *zT* (bottom, Figure 3B).

For the additional heat absorption (middle, Figure 3C), the Seebeck coefficient at the cold end should be pre-designed to be lower than the optimal Seebeck coefficient while that at the hot end should be pre-designed to be higher than the optimal Seebeck coefficient (the chemical potential grading is parallel to the temperature grading, e.g. ∇_*x*_*μ*∇_*x*_*T*>0). This ensures the increase in electronic entropy throughout the leg, though the achievable average *zT* remains bounded below the peak *zT* (bottom, Figure 3C). To simplify the analysis, we adopt the *zT*_avg._ value as an approximation for the integrated average, as indicated in Figure 3A. This approach challenges the conventional understanding of graded materials in thermoelectric cooling.

Accordingly, one can compare the cooling performance between these two approaches (∇_*x*_*μ*∇_*x*_*T* < 0 for the optimal *zT* with *zT*_peak_ and ∇_*x*_*μ*∇_*x*_*T* > 0 for the additional heat absorption with *zT*_ave._). The relative enhancement in the cooling performance is quantified by (Equation 12a), (Equation 12b) for different *S*_h_*/S*_c_ and *B* factors (Figure 3D). The results indicate that there would be an optimal *S*_h_*/S*_c_ for the extended Peltier-enhanced cooling. This reflects the trade-off behavior between maximizing the additional heat absorption and preserving the material’s *zT*. More inspiringly, a smaller *B* factor (e.g., high lattice thermal conductivity at low temperatures) can lead to more significant improvements. This indicates that the extended Peltier effect holds particular promise for cryogenic thermoelectric cooling, as low *B* factors are more readily achieved at cryogenic temperatures.

## Conclusion

In summary, this work utilizes the nonreciprocal thermoelectric cooling effect, which involves all independent variables that can cause a rapid increase in the Seebeck coefficient, including temperature (*T*) and chemical potential (*μ*). To verify its effectiveness, n-type Bi_2_Te_2.7_Se_0.3_ single crystals with a spatially graded Seebeck coefficient were experimentally synthesized by controlling the composition gradient. Single-leg cooling devices are fabricated to extract the contribution of the extended Peltier effect to cooling performance. The cooling performance measurements reveal that Δ*S*_*x*_ of ∼50 μV·K^−1^ results in a 10% improvement in maximum temperature span compared to the traditional Peltier effect. The observations are consistent with the predictions due to the nonreciprocal cooling temperature span, further validating the proposed framework. The strategy proposed in this work can inspire diverse strategies for manipulating the generalized thermoelectric cooling effect, especially for cryogenic applications.

## Resource availability

### Materials availability

Materials fabricated in this study are available from the corresponding author upon reasonable request and a completed materials transfer agreement.

### Data and code availability

The data that support the findings of this study are available from the corresponding author upon reasonable request.

## Funding and acknowledgments

The authors thank Prof. Jeffrey Snyder for valuable discussions. This work is supported by the 10.13039/501100001809National Natural Science Foundation of China (grant nos. T2125008, 52571249, 92263108, and 92163203), the 10.13039/501100013105Shanghai Rising-Star Program (23QA1409300), and the Innovation Program of Shanghai Municipal Education Commission (2021-01-07-00-07-E00096). The funders had no role in the study design, data collection and analysis, decision to publish, or preparation of the manuscript.

## Author contributions

The work was conceived and designed by X.Z., Z.C., J.L., and Y.P. Z.C. and X.Z. deduced the equations of the model. H.Z., S.Z., and X.W. synthesized the materials, fabricated the cooling device, and characterized the performance. X.Z., S.W., M.X., and J.Z. characterized the Seebeck coefficient mapping. H.Z., X.Z., and Z.C. drafted the manuscript. W.L., J.L., and Y.P. revised the manuscript. All authors contributed to the manuscript and approved the final version.

## Declaration of interests

The authors declare no competing interests.

## Supplemental information

Document S1. Figures S1–S10

Document S2. Article plus supplemental information
