# Supplementary material for: Nonreciprocal thermoelectric cooling by spatially grading Seebeck coefficient for cooling enhancement
Source: Innovation (Camb). 2026 May 27;7(11):101438. doi: 10.1016/j.xinn.2026.101438 (PMC13751141; doi:10.1016/j.xinn.2026.101438)
Supplement: Supplementary file 1 [file mmc1.pdf]

The Innovation, Volume *7*

Supplemental Information

Nonreciprocal thermoelectric cooling by spatially grading Seebeck coefficient for cooling enhancement

Han Zhao, Zhiwei Chen, Xinyue Zhang, Shuxian Zhang, Xuemei Wang, Shaoqin Wang, Minghao Xue, Jiye Zhang, Wen Li, Jun Luo, and Yanzhong Pei

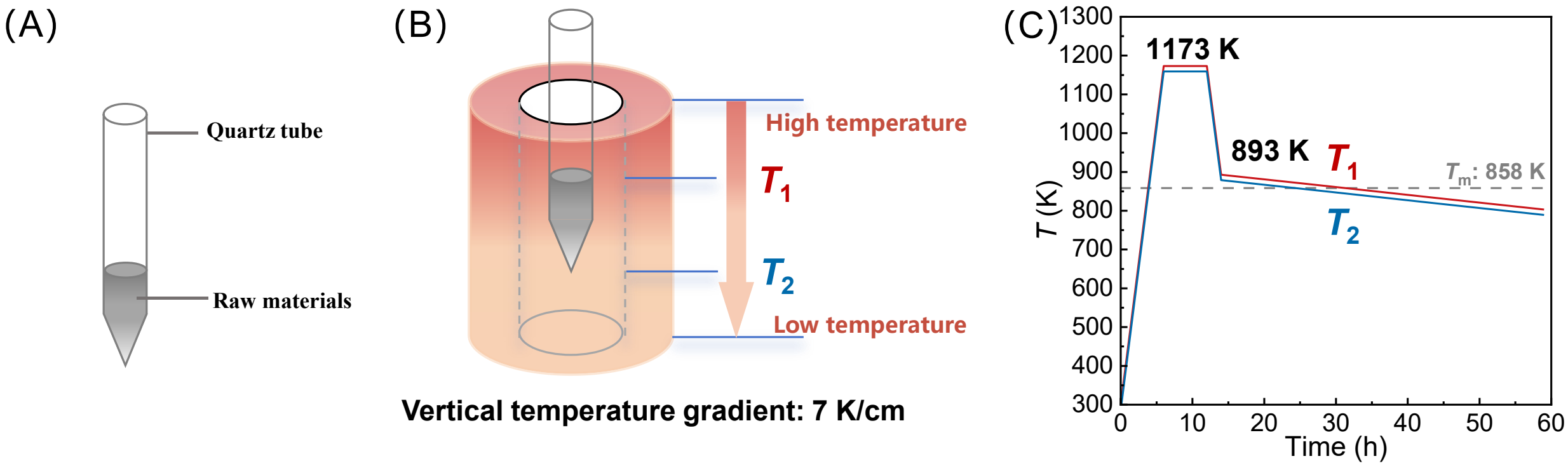

Figure S1. Schematic diagram of crystals growth. (A) Raw materials were weighed and loaded into a tapered quartz tube; (B) the sealed quartz tube was placed in a tube furnace with a temperature gradient of 7 K/cm; (C) Temperature control set during the growth process.

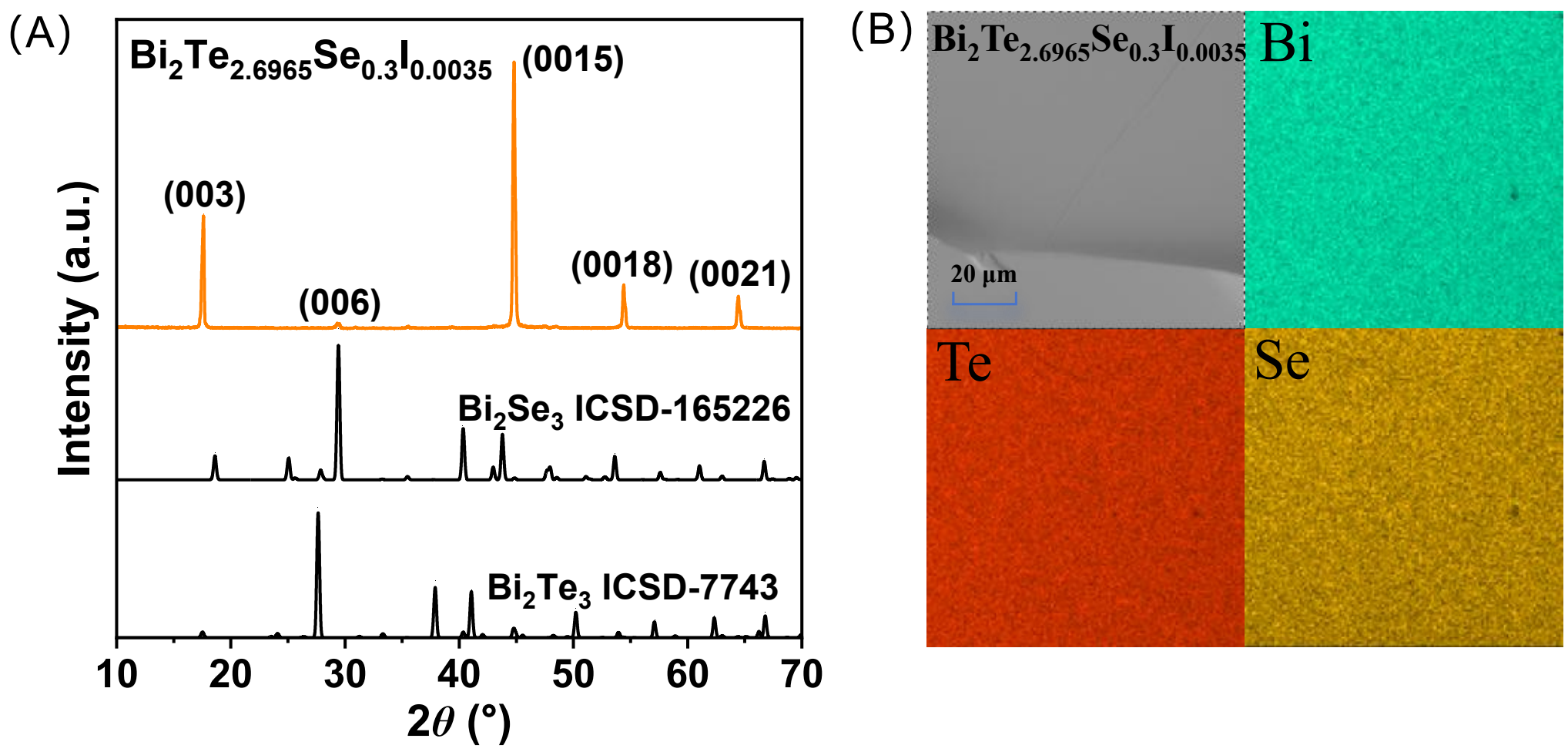

Figure S2. Phase characterization. (A) XRD patterns and (B) SEM image and EDS mapping for $Bi_2Te_{2.6965}Se_{0.3}I_{0.0035}$.

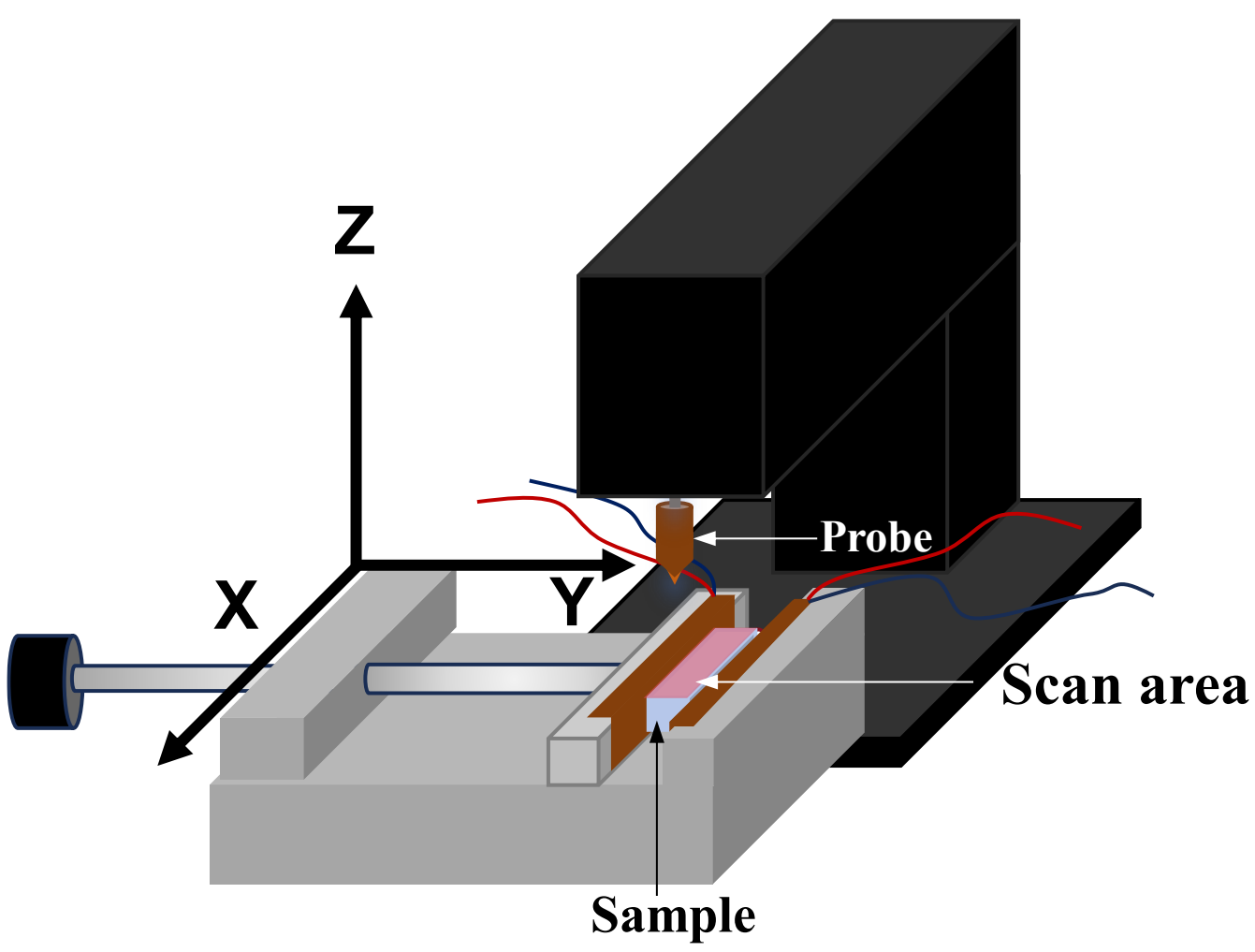

Figure S3. Schematic diagram of the setup for Seebeck coefficient mapping using PSM II.

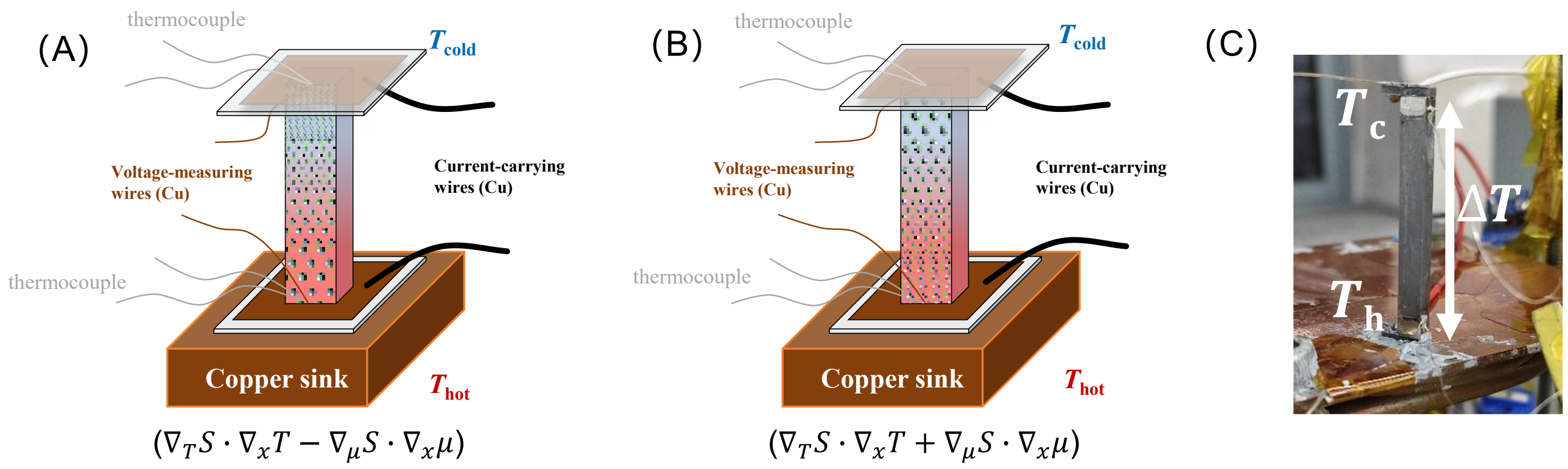

Figure S4. Schematic diagram of cooling and Harman *ZT* measurement on a n-type material. (A) Current flows from the low |*S*| end, where the net cooling combines the traditional Peltier effect minus the extended Peltier effect (‘$P_{trad.}$-$P_{ext.}$’ mode). (B) Current flows from the high |*S*| end, where the net cooling combines the traditional Peltier and the extended Peltier effects (‘$P_{trad.}$+$P_{ext.}$’ mode). (C) A photograph of the measurement setup.

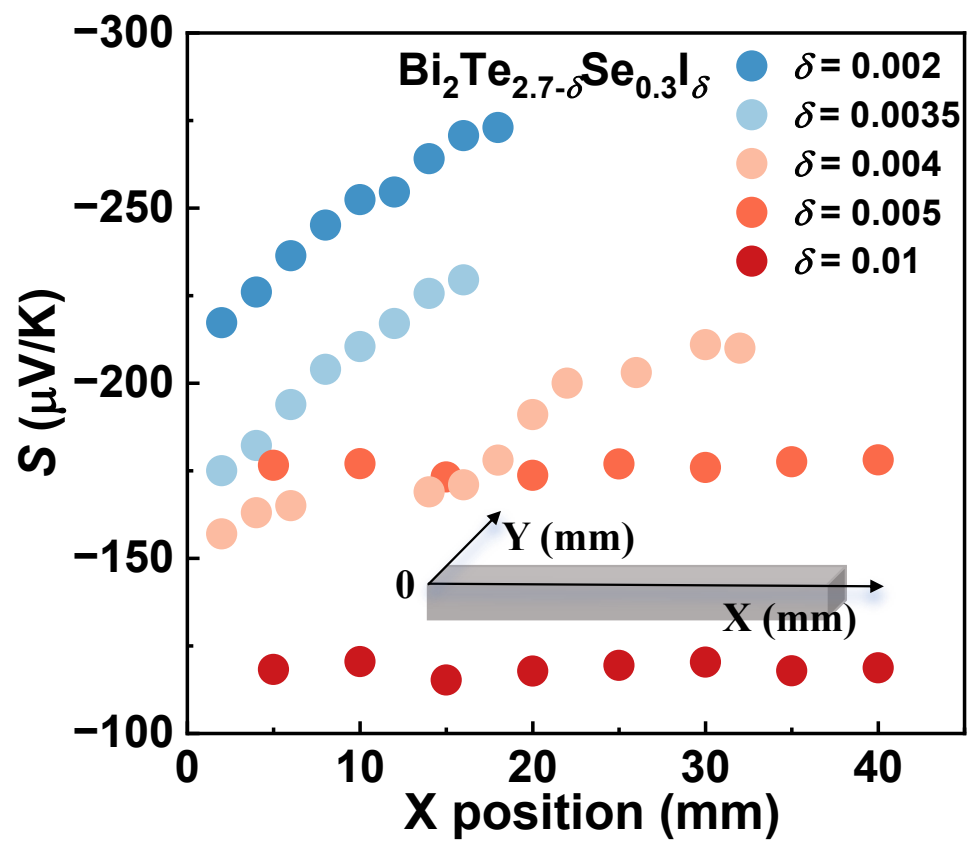

Figure S5. Seebeck coefficient distribution for $Bi_2Te_{2.7-\delta}Se_{0.3}I_\delta$.

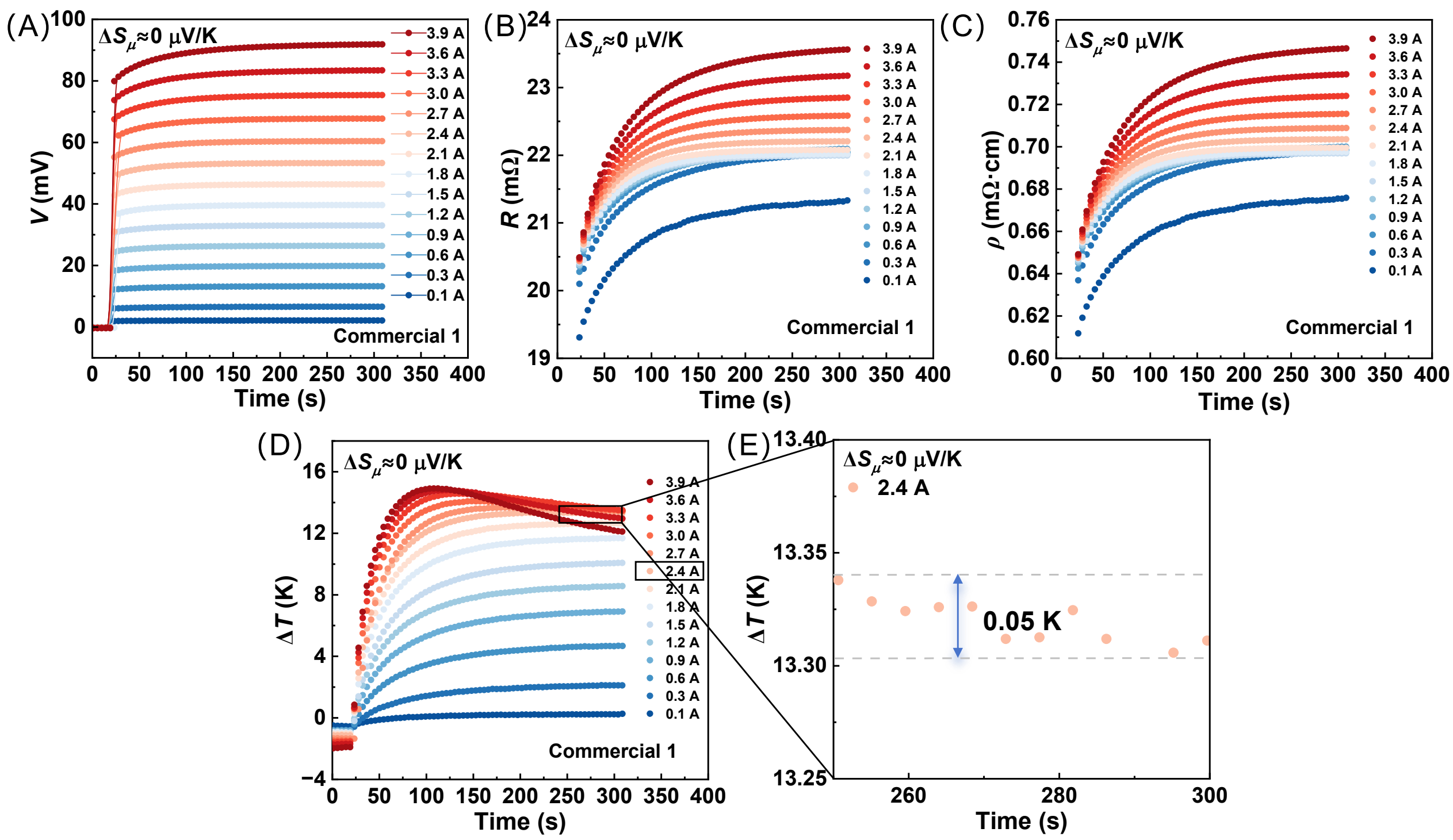

Figure S6. Measurement of Harman *ZT* and temperature span (ΔT) at ‘$P_{trad.}$+$P_{ext.}$’ mode for $\Delta S_\mu \approx 0$ μV/K. (A) Voltage, (B) resistance, (C) resistivity and (D) temperature span (ΔT) as a function of time for $\Delta S_x \approx 0$ μV/K sample. (D) Magnified view of the stabilized ΔT versus time, showing that the fluctuation is within 0.05 K.

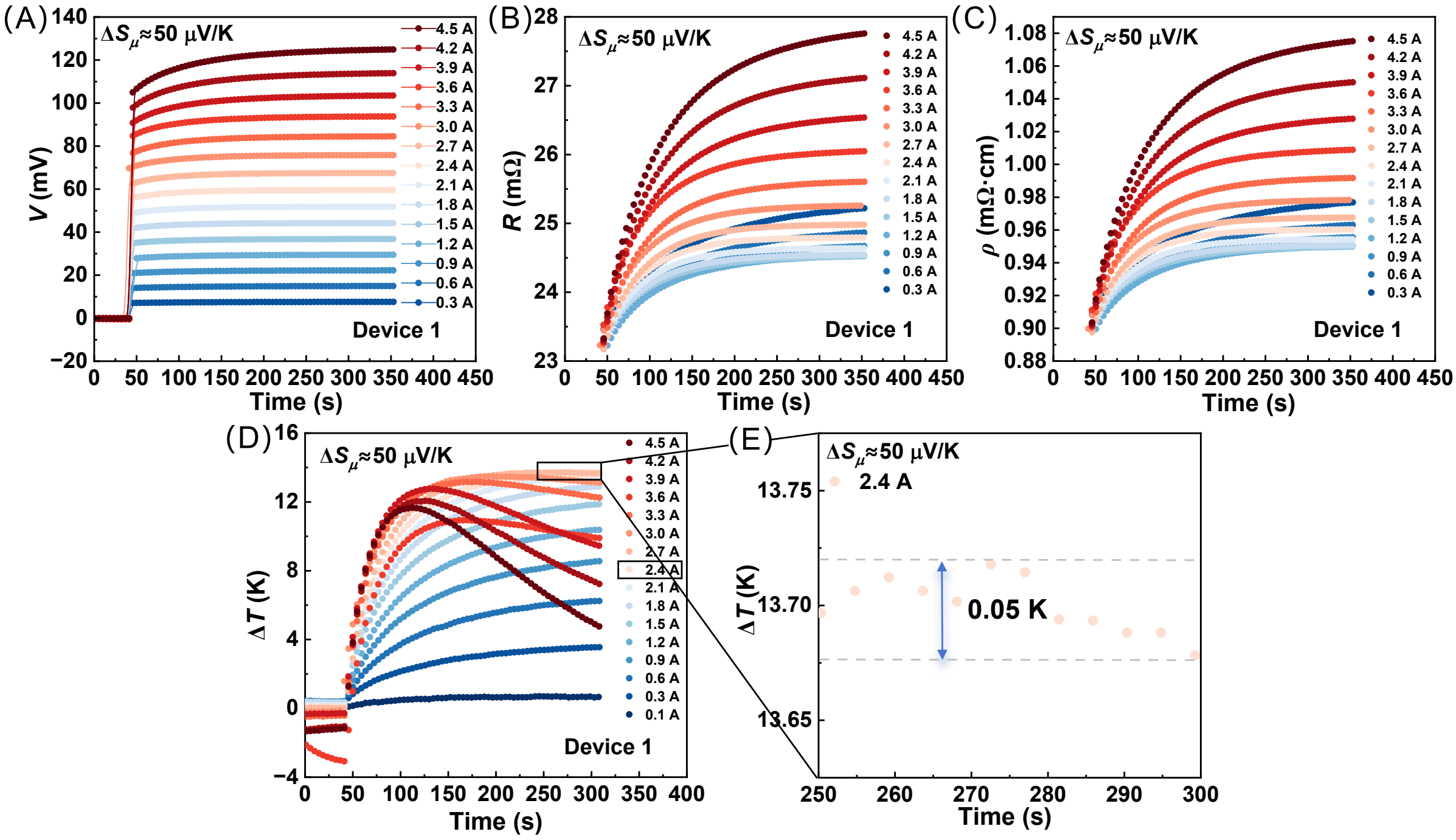

Figure S7. Measurement of Harman *ZT* and temperature span (ΔT) at '$P_{trad.}$+$P_{ext.}$' mode for $\Delta S_\mu \approx 50$ μV/K. (A) Voltage, (B) resistance, (C) resistivity and (D) temperature span (ΔT) as a function of time for $\Delta S_x \approx 50$ μV/K sample. (D) Magnified view of the stabilized ΔT versus time, showing that the fluctuation is within 0.05 K.

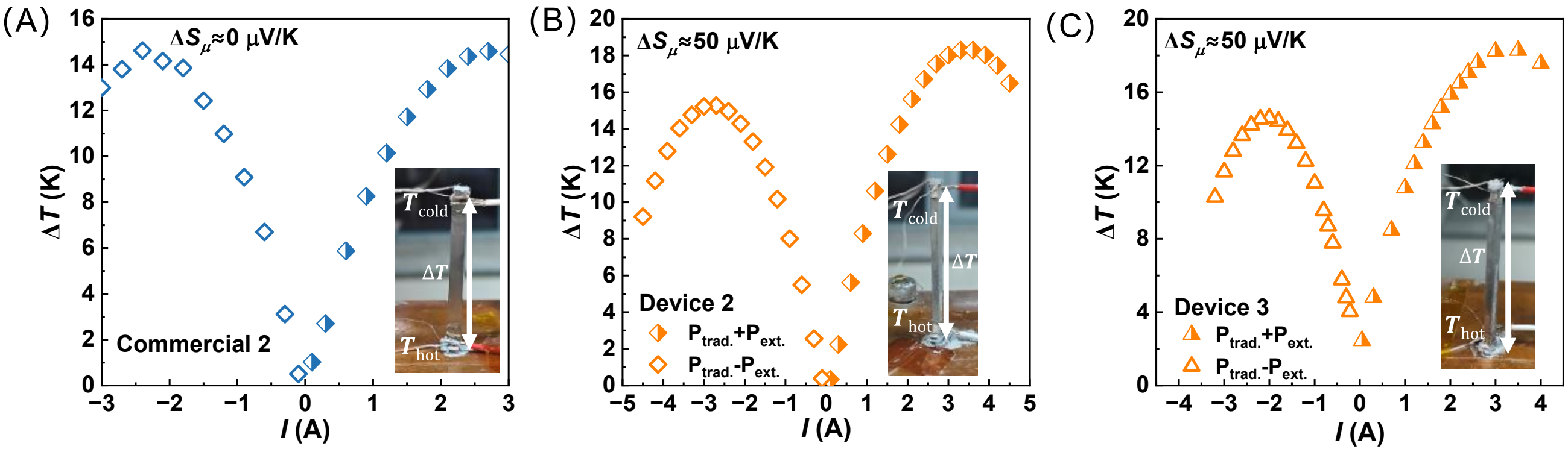

Figure S8. Cooling performance for commercial and graded n-type $Bi_2Te_{2.7}Se_{0.3}$ alloys. Current dependent temperature span (ΔT) for (A) commercial 2, (B) compositionally graded device 2 and (B) compositionally graded device 3.

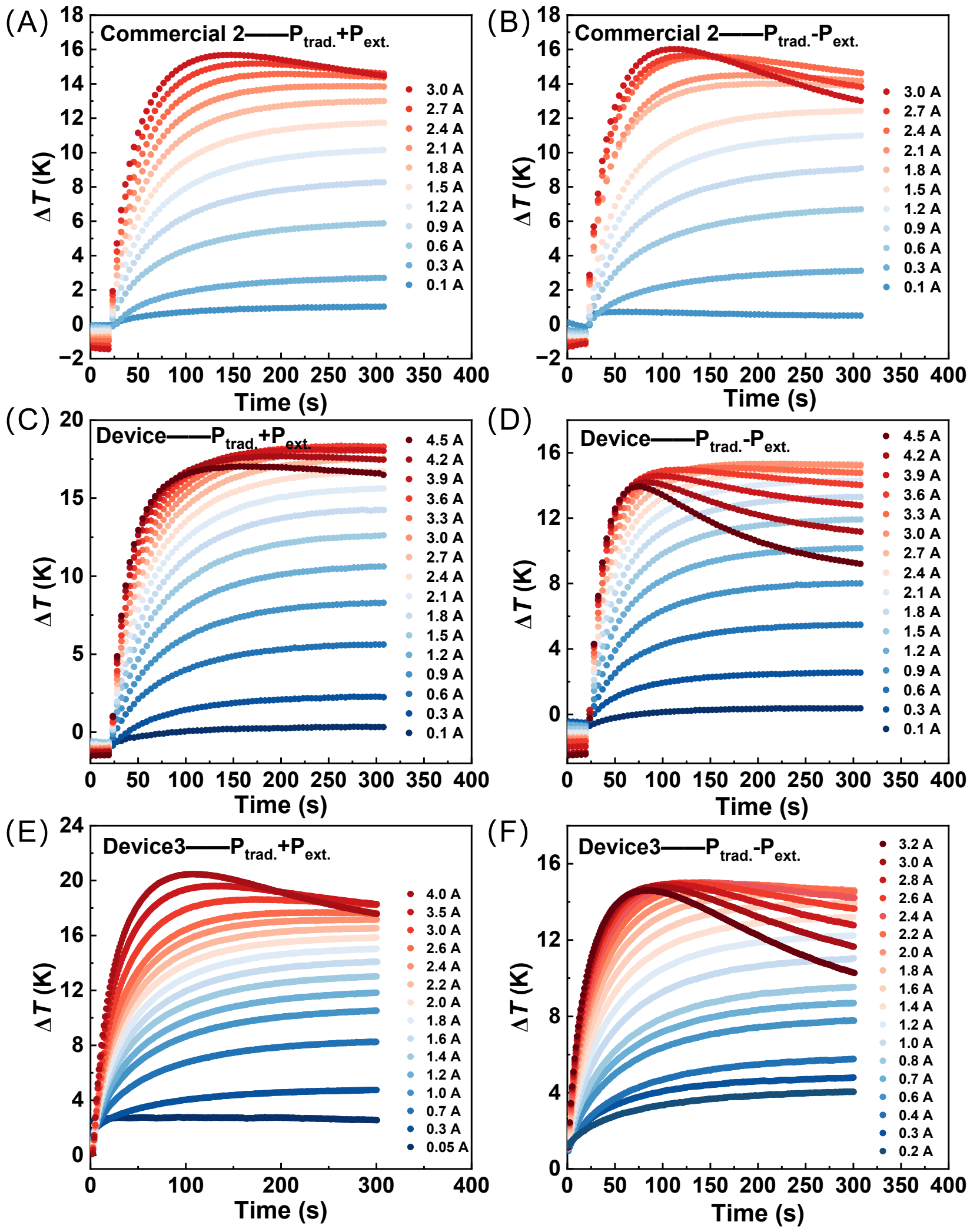

Figure S9. Temperature span versus time at different current. (A) Temperature span (ΔT) versus time at '$P_{trad.}$+$P_{ext.}$' mode for commercial 2; (B) ΔT versus time at '$P_{trad.}$-$P_{ext.}$' mode for commercial 2; (C) ΔT versus time at '$P_{trad.}$+$P_{ext.}$' mode for graded device 2; (D) ΔT versus time at '$P_{trad.}$-$P_{ext.}$' mode for graded device 2; (E) ΔT versus time at '$P_{trad.}$+$P_{ext.}$' mode for graded device 3; (F) ΔT versus time at '$P_{trad.}$-$P_{ext.}$' mode for graded device 3.

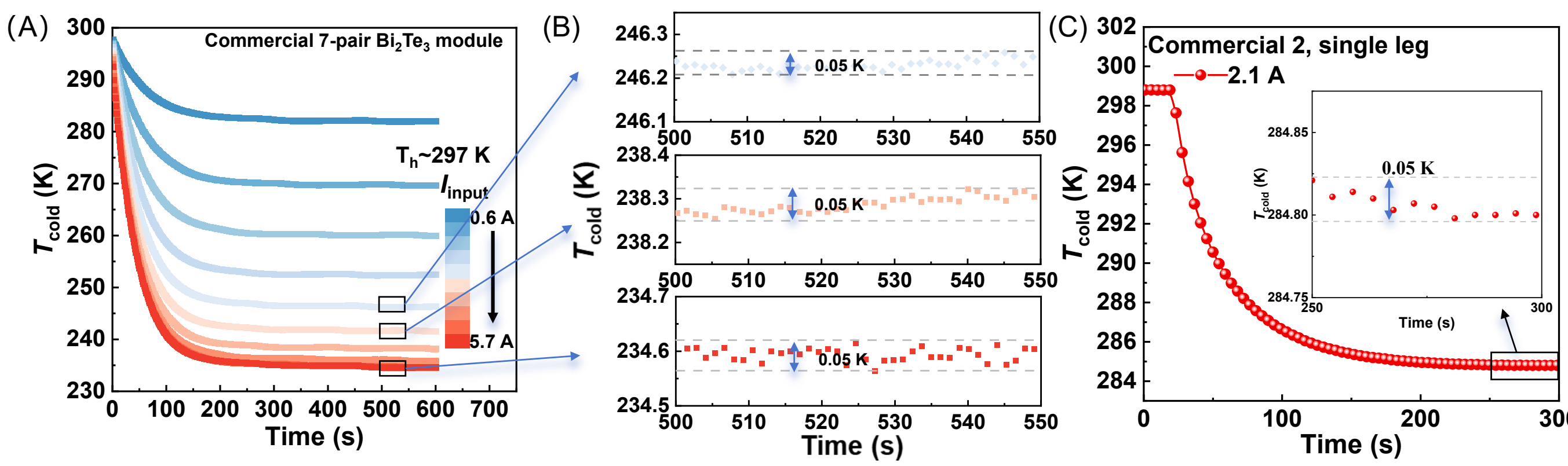

Figure S10. Temperature measurement accuracy of the test system. (A) Temperature span (ΔT) versus time at different current for a commercial 7-pairs Bi_2Te_3 module. (A) Magnified view of the stabilized ΔT as a function of time, showing the temperature fluctuation is within 0.05 K. (C) ΔT versus time for a commercial extruded n-type single leg device, the inset of which also shows a temperature fluctuation within 0.05 K.
